# Isolated Testicular Tuberculosis Disguised As a Testicular Tumor: A Report of a Rare Case

**DOI:** 10.7759/cureus.60763

**Published:** 2024-05-21

**Authors:** Deerush Kannan Sakthivel, Madhav Tiwari, Sandeep Bafna, Narasimhan Ragavan

**Affiliations:** 1 Urology, Apollo Hospitals, Chennai, IND

**Keywords:** tuberculosis, genital tuberculosis, testicular cancer, testicular tuberculosis, testicular mass

## Abstract

Isolated testicular tuberculosis is rare, often diagnosed incidentally during histopathological examination due to its asymptomatic nature. We present a case of a 35-year-old male with a left testicular mass mimicking malignancy. Despite normal tumor markers and negative imaging for pulmonary tuberculosis, left inguinal orchiectomy revealed testicular tuberculosis. Diagnostic challenges are compounded by the disease's rarity and atypical presentation. Genitourinary tuberculosis's diagnostic complexity underscores the need for heightened clinical suspicion, particularly in tuberculosis-endemic regions. While orchiectomy may be necessary, this case underscores the importance of considering tuberculosis in testicular masses. Early recognition facilitates appropriate management and underscores the importance of diagnostic vigilance.

## Introduction

Isolated testicular tuberculosis without evidence or symptoms elsewhere is an extremely rare condition [1]. The diagnosis can be challenging especially when there are no associated symptoms in a healthy individual. Owing to the rarity of the condition and complexity of the presentation, it is likely to be picked up at the time of histopathological examination [1]. Though there are novel diagnostic tests for diagnosis and drug susceptibility to improve treatment outcomes, early diagnosis of tuberculosis is crucial and it can be only possible when the clinician has a working diagnosis of tuberculosis as one of the differentials [2].

## Case presentation

**Figure 1:**
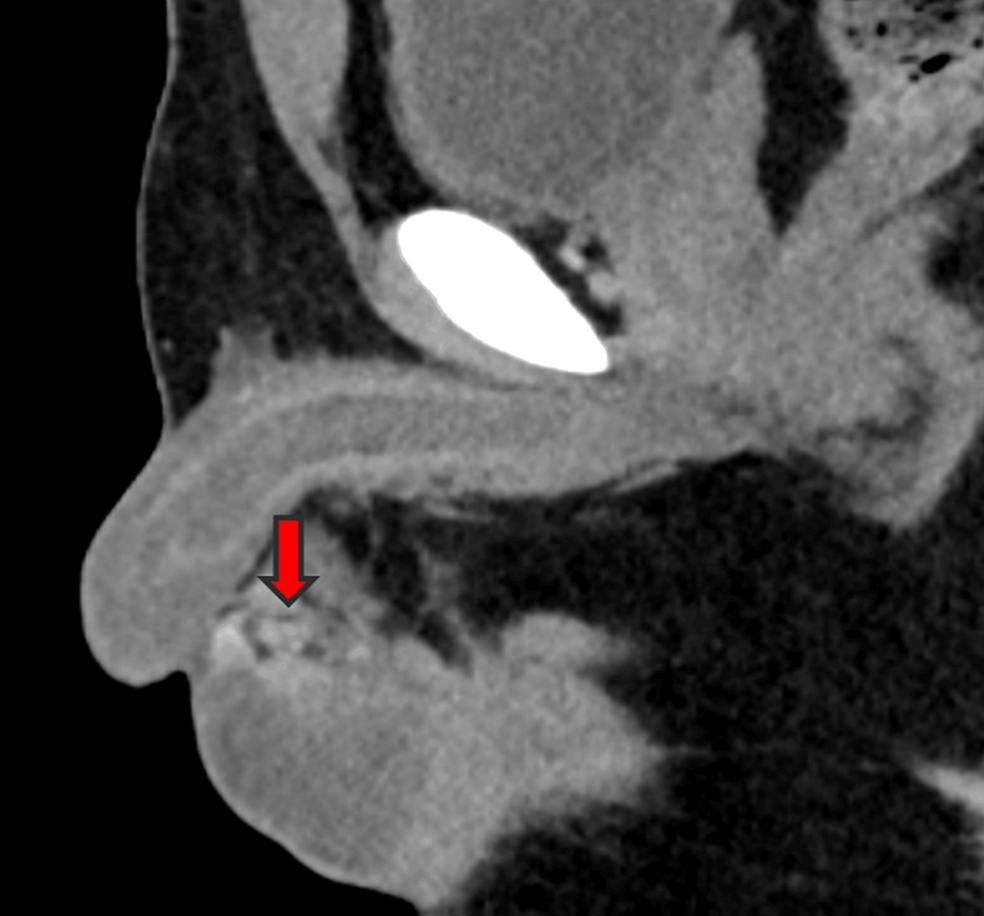
Enhancing solid nodular lesion in the upper pole of the testis

**Figure 2:**
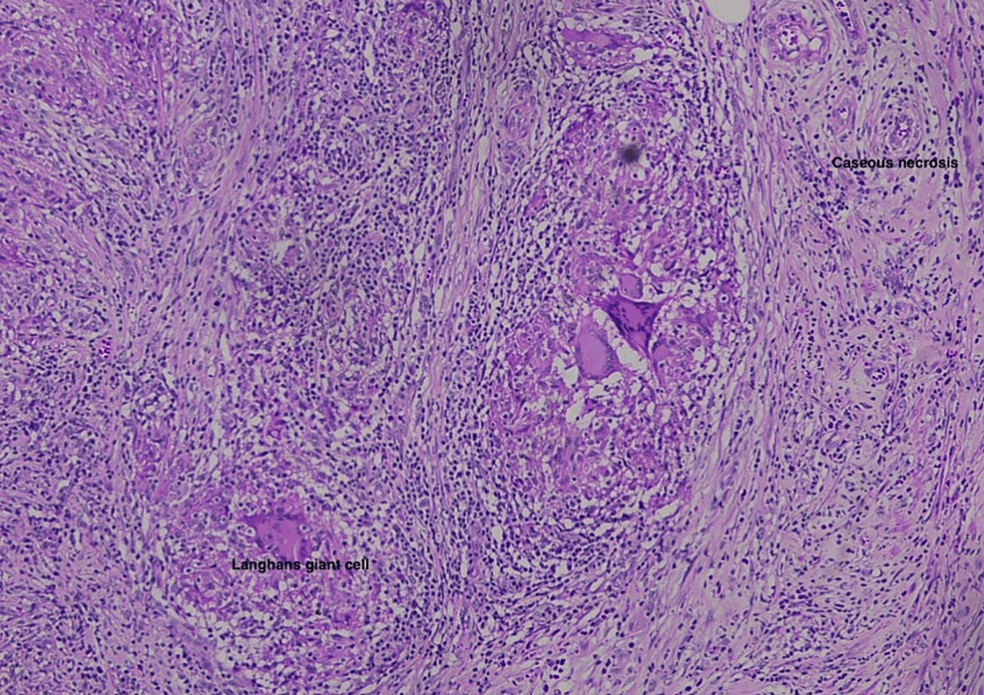
Pathological image showing a Langhans giant cell and caseous necrosis

We report herein the case of a 35-year-old gentleman from India who presented with a left testicular mass. He had no significant medical history and no family history of tuberculosis. Apart from the testicular mass, there were no associated symptoms of night sweats or weight loss. A firm mass was palpable in the upper pole of the testis. Ultrasound examination was suggestive of a 2 cm hypervascular, homogenous, and hypoechoic mass in the upper pole of the left testis with a normal appearance of the epididymis and vas. Given the ultrasound findings, a CT abdomen with contrast was performed subsequently, which confirmed an enhancing solid nodular lesion adjacent to the upper pole of the testis, and there was no evidence of retroperitoneal lymphadenopathy (Figure 1). Since the only working diagnosis was a testicular tumor, markers were done and were within normal range. As a part of the surgical workup, a chest X-ray was done that did not reveal any evidence of pulmonary tuberculosis. In view of the presentation and the imaging findings pointing toward a malignancy, it was decided to proceed with a left inguinal orchiectomy. Surprisingly, the pathological analysis uncovered tuberculosis affecting the testis (Figure 2). The clinical presentation closely resembled that of testicular cancer, lacking distinctive signs suggestive of tuberculosis. 

## Discussion

Genitourinary tuberculosis (GUTB) is one of the common presentations of extrapulmonary tuberculosis. Following a focus in the lungs, GUTB occurs in 2-20% of patients as a result of the bloodstream spread of infection to the kidneys, prostate, and epididymis. It can also travel through the descending collecting system to the ureters, bladder, and urethra, as well as through the ejaculatory ducts to the genital organs [3].

A rare case of isolated urinary bladder tuberculosis has been reported, however, the patient had nonspecific constitutional symptoms and contact history with a patient with pulmonary tuberculosis and persistent sterile pyuria [4]. In our case, since it was an isolated lesion in the testis, the chances of urine routine showing pyuria could not be expected and the Gene Xpert test of the urine sample was also negative when it was done in the postoperative period.

In a retrospective study on 117 patients with GUTB, there were no cases of testicular or penile tuberculosis reported over a period of 13 years [5]. In the same study, the diagnostic positivity rate was 41.6% for the urine acid-fast bacilli (AFB) test, 55.4% for the urine Mycobacterium tuberculosis culture test, and 67.7% for polymerase chain reaction (PCR). Chest X-ray was positive in 25.6%, erythrocyte sedimentation rate (ESR) was raised in 62.5%, and the Mantoux test was positive in 61.2% of patients [5]. In a relatively large proportion of the cohorts, not a single test had a diagnostic positivity rate of more than 70%. This very well highlights the lacunae in the diagnostic modalities for the condition.

Adding to the diagnostic difficulties and the varied presentations, it could be a hard fact to note that GUTB remains considerably less understood compared to prevalent urological conditions such as urolithiasis or uro-oncological disorders. Despite indicative symptoms like hematuria, sterile pyuria, and recurrent urinary tract infections, the diagnostic consideration for GUTB is often overlooked. This oversight may stem from the absence of universally recognized guidelines, a consequence of the disease's endemic distribution. Therefore, a deeper understanding of the clinical features of urogenital tuberculosis is imperative, underscoring the significance of early detection. Though there has been a previous presentation of a patient very similar to this case, an orchidectomy was performed by the authors owing to the same complexities associated with the condition but the corresponding histopathology revealed a pus pocket with caseous necrosis [6].

It is crucial to keep tuberculosis in mind as part of the differential diagnosis and to promptly refer patients to an infectious disease specialist or physician when there is uncertainty regarding treatment and follow-up. This emphasis is particularly important in clinical practices based on large-scale populations in India.

## Conclusions

Our presentation aims not to debate the avoidability of orchidectomy but to highlight the infrequency of testicular involvement in the disease. We also emphasize the necessity of considering tuberculosis as a potential differential diagnosis for testicular tumors, especially in regions where the disease is common. This could potentially save the time to diagnosis and avoid unnecessary investigations and a surprise diagnosis of tuberculosis after an orchidectomy.
